# Traditional Korean Herbal Formula Samsoeum Attenuates Adipogenesis by Regulating the Phosphorylation of ERK1/2 in 3T3-L1 Cells

**DOI:** 10.1155/2015/893934

**Published:** 2015-09-21

**Authors:** Soo-Jin Jeong, Sae-Rom Yoo, Chang-Seob Seo, Hyeun-Kyoo Shin

**Affiliations:** ^1^KM Convergence Research Division, Korea Institute of Oriental Medicine, Daejeon 305-811, Republic of Korea; ^2^Korean Medicine Life Science, University of Science & Technology, Daejeon 305-350, Republic of Korea; ^3^K-Herb Research Center, Korea Institute of Oriental Medicine, Daejeon 305-811, Republic of Korea

## Abstract

Adipogenesis is the cell differentiation process from preadipocytes into adipocytes and the critical action in the development of obesity. In the present study, we conducted *in vitro* analyses to investigate the inhibitory effects of Samsoeum (SSE), a traditional herbal decoction. SSE had no significant cytotoxic effect against either the undifferentiated or differentiated 3T3-L1 cells. Oil Red O staining results showed that SSE significantly inhibited fat accumulation in adipocytes. SSE treatment consistently reduced the intracellular triglyceride content in the cells. SSE significantly inactivated glycerol-3-phosphate dehydrogenase (GPDH), a major link between carbohydrate and lipid metabolisms in 3T3-L1 adipocytes, and markedly inhibited the production of leptin, an important adipokine, in differentiated cells. SSE markedly suppressed the mRNA expression of the adipogenesis-related genes peroxisome proliferator-activated receptor-gamma (*PPAR-γ*), CCAAT/enhancer binding protein-alpha (*C/EBP-α*), fatty acid synthase (*FAS*), lipoprotein lipase (*LPL*), and fatty acid binding protein 4 (*FABP4*). Importantly, SSE increased the phosphorylation of ERK1/2, but not p38 MAPK and JNK, in adipose cells. Overall, our results indicate that SSE exerts antiadipogenic activity and modulates expressions of adipogenesis-related genes and ERK1/2 activation in adipocytes.

## 1. Introduction

Obesity is a major contributor to the development of type 2 diabetes, hyperglycemia, and cancer. Along with diet, exercise, behavior modification, and medications are used in the treatment of overweight or obese people. Among these treatments, medications are an important part of the treatment process for morbid obesity, but weight-loss drugs can have serious side effects. Sibutramine and orlistat have been used in the treatment of obesity for the past two decades. However, sibutramine, which suppresses the appetite, increases the risk of heart attack and stroke in patients with a history of cardiovascular disease [1, 2]. Orlistat also has side effects including steatorrhea, dark urine, and stomach pain [2, 3]. To overcome these limitations, a new approach to the treatment of obesity has been developed using complementary and alternative medicines such as herbal medicines.

Samsoeum (SSE,* Shensuyin* in Chinese,* Jinsoin *in Japanese), a traditional herbal medicine, was first recorded during the Song dynasty (China, AD 1107). SSE comprises 12 medicinal herbs including* Perilla frutescens, Pueraria lobata, Pinellia ternata, Angelica decursiva, Panax ginseng, Poria cocos, Citrus aurantium, Platycodon grandiflorum, Glycyrrhiza uralensis, Citrus unshiu, Zingiber officinale,* and* Zizyphus jujuba*. It is used to treat the common cold, fever, and headache. Studies in recent years have established that SSE has pharmacological properties such as immune regulation, anticancer, and anti-inflammation [4–6]. SSE can also prevent allergic reactions after exposure to allergens and therefore may be an antiallergic agent [7, 8]. Despite these observations, few scientific studies have examined its antiobesity effect.

In the present study, we evaluated inhibitory effect of SSE on 3T3-L1 adipocytes treated with SSE. We also investigated its mechanisms of action by examining its effects on the expressions of genes and proteins involved in lipid metabolisms.

## 2. Materials and Methods

### 2.1. Plant Materials

The 12 herbal medicines forming SSE were purchased from Omniherb (Yeongcheon, Korea) and HMAX (Jecheon, Korea). The origin of these herbal medicines was taxonomically confirmed by Professor Je Hyun Lee (Dongguk University, Gyeongju, Korea). A voucher specimen (2008–KE28–1~KE28–12) has been deposited at the K-herb Research Center, Korea Institute of Oriental Medicine.

### 2.2. Preparation of SSE Water Extract

SSE decoction comprising the 12 herbal medicines including Perillae Folium, Puerariae Radix, Pinelliae Tuber, Angelicae Decursive Radix, Ginseng Radix Alba, Poria Sclerotium, Aurantii Fructus Immaturus, Platycodonis Radix, Glycyrrhizae Radix et Rhizoma, Citri Unshius Pericarpium, Zingiberis Rhizoma Crudus, and Zizyphi Fructus was mixed (Table 1; 3.5 kg; 41.25 g × 85) and extracted in a 10-fold mass of water at 100°C for 2 h under pressure (1 kgf/cm^2^) using an electric extractor (COSMOS-660; Kyungseo Machine Co., Incheon, Korea). The water extract was then filtered through a standard sieve (number 270, 53 *μ*m; Chung Gye Sang Gong Sa, Seoul, Korea), and the solution was evaporated to dryness and freeze dried to give a powder. The yield of SSE water extract was 18.6% (651.4 g).

### 2.3. Cell Culture and Differentiation

The mouse 3T3-L1 preadipocyte cell line was obtained from the American Type Culture Collection (CL-173, ATCC, Rockville, MD). The cells were cultured in DMEM (Gibco BRL, Carlsbad, CA) supplemented with 10% newborn calf serum (Gibco BRL, Carlsbad, CA) at 37°C. For adipocyte differentiation, the cells were stimulated with 3T3-L1 differentiation medium containing isobutylmethylxanthine, dexamethasone, and insulin (MDI) (Zen-Bio Inc., Research Triangle Park, NC) for 48 h after reaching a confluent state. The medium was switched to DMEM containing 10% FBS and 1 *μ*g/mL insulin after 2 days and then changed to DMEM containing 10% FBS for an additional 4 days. SSE extract was added to the cell culture during the 8 days of differentiation. GW9662 (Sigma-Aldrich, St. Louis, MO), PPAR-*γ* antagonist, was used as positive control.

### 2.4. Cytotoxicity Assay

Undifferentiated 3T3-L1 cells were treated with various concentrations of SSE for 24 h. To produce differentiated adipocyte cells, 3T3-L1 preadipocytes were differentiated for 8 days by stimulating them by SSE. CCK-8 solution (Dojindo, Kumamoto, Japan) was added, and the cells were incubated for 4 h. After incubation, the absorbance was read at 450 nm on a microplate reader (Benchmark Plus, Bio-Rad. Hercules, CA).

### 2.5. Oil Red O Staining

The differentiated 3T3-L1 cells were fixed with 10% formalin for 15 min at room temperature and washed with 70% ethanol and PBS. The cells were stained with Oil Red O (Sigma-Aldrich, St. Louis, MO) for 5 min and then washed with PBS. Cell images were collected using an Olympus CKX41 inverted microscopy (Olympus, Tokyo, Japan). Stained oil droplets were dissolved in isopropyl alcohol and measured by reading the absorbance at 520 nm using microplate reader (Benchmark Plus, Bio-Rad. Hercules, CA).

### 2.6. Triglyceride Quantification Assay

The triglyceride concentration was measured enzymatically using a commercial kit (BioVision Inc., Milpitas, CA). Briefly, the 3T3-L1 adipocytes treated with SSE were homogenized in 5% NP-40 assay buffer and the sample to solubilize all triglycerides. The sample was mixed with lipase and triglyceride reaction mixture. After a 1 h incubation, the sample absorbance was measured at 570 nm using microplate reader (Benchmark Plus, Bio-Rad. Hercules, CA).

### 2.7. Glycerol-3-Phosphate Dehydrogenase (GPDH) Activity Assay

After the induction of adipocyte differentiation with treating with SSE, 3T3-L1 cells were washed twice with PBS. GPDH activity was measured using a commercial kit (TAKARA, Tokyo, Japan) and by monitoring the dihydroxyacetone phosphate-dependent oxidation of NADH at 340 nm. GPDH activity was expressed as unit/mg of protein.

### 2.8. Leptin Immunoassay

Leptin concentration was measured using a mouse leptin immunoassay kit (R&D Systems, Minneapolis, MN) according to the manufacturer's instructions. In brief, the culture supernatant was collected from the differentiated 3T3-L1 adipocytes that has been treated with or without SSE. Equal amounts of the supernatants (50 *μ*L) and Assay Diluent RD1W (50 *μ*L) were added to a 96-well plate, and the plate was incubated for 2 h at room temperature. The plates were washed 5 times with 400 *μ*L of wash buffer; 100 *μ*L of mouse leptin conjugate was added to each well and incubated for 2 h at room temperature. The plates were washed 5 times; 100 *μ*L of substrate solution was added to each well and incubated for 30 min at room temperature in the dark. Finally, 100 *μ*L of stop solution was added to each well, and the absorbance was measured at 450 nm using microplate reader (Benchmark Plus, Bio-Rad. Hercules, CA).

### 2.9. RNA Isolation and Real-Time RT-PCR

Total RNA was prepared using TRIzol reagent (Invitrogen, Carlsbad, CA). Real-time RT-PCR analysis was performed using an Applied Biosystems 7300 Real-time PCR system and the SYBR green fluorescence quantification system (Applied Biosystems, Foster City, CA) to quantify the amplicons. cDNA was synthesized using 100 ng of RNA in a reverse transcription reaction. The PCR conditions were 50 cycles of 95°C (30 s), 55°C (30 s), and a standard denaturation curve. The primer sequences are listed in the 5′ to 3′ orientation in Table 2. The PCR conditions for each target were optimized according to the primer concentration, the absence of primer dimer formation, and the efficiency of amplification of both the target genes and the housekeeping control gene. PCR reactions mixture comprised 1 *μ*L of cDNA and 9.5 *μ*L of PCR master mix, which contained 2x SYBR Green, 10 pmole each of the forward and reverse primer, and 4.5 *μ*L of DEPC-treated distilled water in a final volume of 15 *μ*L. To normalize the cDNA content of the samples, we used the comparative threshold (C_T_) cycle method, which includes normalization of the number of target gene copies versus the endogenous reference gene, GAPDH. The C_T_ is defined as the fractional cycle number at which the fluorescence generated by cleavage of the probe passes a fixed threshold baseline when amplification of the PCR products is first detected.

### 2.10. Western Blotting

The protein was extracted using Mammalian Cell Lysis Buffer (Sigma-Aldrich, St. Louis, MO) containing protease inhibitor cocktail (Roche Applied Science, Indianapolis, IN). The protein concentration was measured using a protein assay reagent (Bio-Rad Lab, Hercules, CA). The proteins were resolved by 8–12% SDS-PAGE gels and transferred to polyvinylidene fluoride membranes (Millipore, Billerica, MA). Nonspecific biding sites were blocked with 5% (w/v) skim milk, and the membranes were incubated with the primary antibodies anti-phospho-p38, phospho-JNK, phospho-ERK1/2 (Cell Signaling Tech., Danvers, MA), and *β*-actin (Santa Cruz Biotechnology, Santa Cruz, CA). The membranes were washed three times in TBST and then incubated with secondary antibodies for 1 h at room temperature. Protein expression was detected by using an ECL system (Thermo Scientific, Rockford, IL).

### 2.11. Chemicals and Reagents for HPLC Analysis

Puerarin, daidzin, liquiritin, naringin, glycyrrhizin (all purity ≥98.0%), and hesperidin (purity ≥92.0%) were purchased from Wako Pure Chemical Industries, Ltd. (Osaka, Japan). Neohesperidin (purity ≥99.0%) was obtained from ChromaDex (Irvine, CA). The HPLC-grade reagents methanol, acetonitrile, and water were obtained from J. T. Baker (Phillipsburg, NJ). Glacial acetic acid was obtained from Merck KGaA (Darmstadt, Germany).

### 2.12. Preparation of Standard and Sample Solutions for HPLC Analysis

A standard stock solution of the 7 compounds puerarin, daidzin, liquiritin, naringin, hesperidin, neohesperidin, and glycyrrhizin was dissolved in methanol at concentration of 1.0 mg/mL. For HPLC analysis, 200 mg of lyophilized SSE extract was dissolved in 20 mL of distilled water, and the solution was filtered through a SmartPor GHP 0.2 *μ*m syringe filter (Woongki Science, Seoul, Korea) and then injected into the HPLC system.

### 2.13. HPLC Analysis of SSE

Analysis of the 7 compounds in SSE sample was performed using a Shimadzu LC-20A HPLC system (Shimadzu Co., Kyoto, Japan) comprising a solvent delivery unit, an online degasser, a column oven, an autosampler, and a PDA detector. The data processor was LCsolution software (version 1.24). The analytical column used was a Gemini C_18_ (250 × 4.6 mm; particle size 5 *μ*m, Phenomenex, Torrance, CA) and maintained at 40°C. The mobile phases comprised 1.0% (v/v) acetic acid in water (A) and 1.0% (v/v) acetic acid in acetonitrile (B). The gradient flow was as follows: 5–70% B for 0–40 min, 70–100% B for 40–45 min, 100% B for 45–50 min, and 100–5% B for 55 min. The flow rate was 1.0 mL/min, and the injection volume was 10 *μ*L. The quantitative analysis of the 7 compounds was performed at 254 nm for puerarin, daidzin, and glycyrrhizin, and at 280 nm for liquiritin, naringin, hesperidin, and neohesperidin.

### 2.14. Statistical Analysis

All data were presented as mean ± standard error of the mean (SEM). Group differences were assessed by one-way ANOVA and Tukey's multiple comparison post hoc test using GraphPad InStat ver.3.10 (GraphPad software Inc., San Diego, CA). The significance of the differences between the sample and normal control at *p* < 0.05 or 0.01 was considered significant.

## 3. Results

### 3.1. Cytotoxic Effects of SSE against Undifferentiated and Differentiated 3T3-L1 Cells

**Figure 1:**
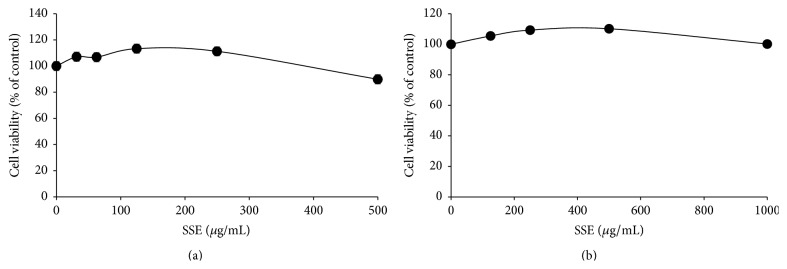
Cytotoxic effects of SSE extract in undifferentiated and differentiated 3T3-L1 cells. (a) 3T3-L1 preadipocytes were treated with various concentrations of SSE (0, 31.25, 62.5, 125, 250, or 500 *μ*g/mL) for 24 h. (b) 3T3-L1 preadipocytes were differentiated into adipocytes by incubation with isobutylmethylxanthine, dexamethasone, and insulin (MDI) for 8 days. The cells were exposed to various concentrations of SSE (0, 62.5, 125, 250, 500, or 1000 *μ*g/mL) during the differentiation period. Cell viability was determined using a CCK-8 assay kit by measuring the absorbance at 450 nm. Data are presented as mean ± SEM.

To determine whether SSE had toxic effect, we used a CCK-8 assay to examine the cytotoxicity of SEE. Both preadipocytes and adipocytes were exposed to a concentration range of 31.5 to 1000 *μ*g/mL. As shown in Figure 1, SSE had no cytotoxic effect against 3T3-L1 preadipocytes (a) and adipocytes (b) compared with untreated cells. Nontoxic concentrations of the test materials were used for the subsequent experiments.

### 3.2. Effects of SSE on Adipogenesis in 3T3-L1 Cells

**Figure 2:**
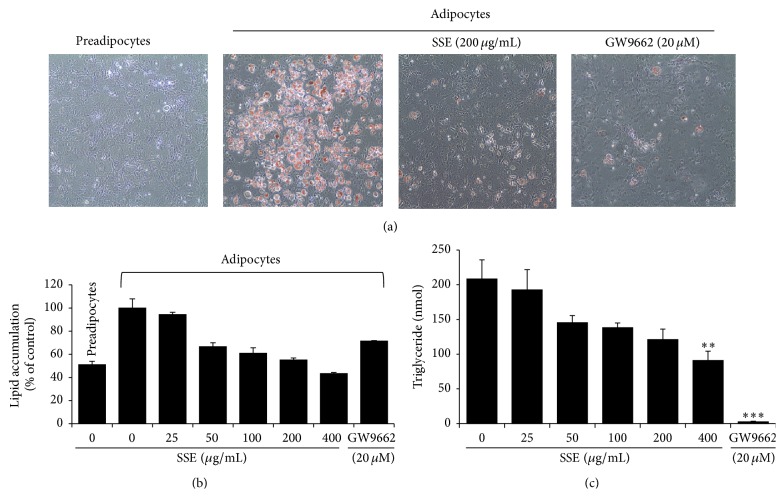
Inhibitory effect of SSE extract on triglyceride production in 3T3-L1 adipocytes. 3T3-L1 preadipocytes were differentiated into adipocytes by incubation with isobutylmethylxanthine, dexamethasone, and insulin (MDI) for 8 days. The cells were treated with or without SSE or GW9662 (20 *μ*M) during the differentiation period. ((a) and (b)) Lipid accumulation in the cells was analyzed by Oil Red O staining. (a) The stained cells were visualized on an Olympus CKX41 inverted microscopy at ×200 of magnification. (b) Stained oil droplets were dissolved in isopropyl alcohol and quantified by reading the absorbance at 520 nm. (c) The triglyceride content was measured enzymatically using a commercial kit (BioVision Inc.) at 570 nm. Data are presented as mean ± SEM. ^*∗∗*^ *p* < 0.01 and ^*∗∗∗*^ *p* < 0.001* versus* differentiated cells.

During adipogenesis, triglycerides are stored in the form of lipid droplets in adipocytes [9]. We used Oil Red O staining to examine the effects of SSE on lipid droplet accumulation. As shown in Figure 2, the number of lipid droplets increased markedly after differentiation for 8 days. Compared with the differentiated control cells, SSE-treated cells had significantly less intracellular lipid droplet accumulation (Figure 2(a)). To quantify the level of lipid accumulation, we dissolved the stained droplets in isopropyl alcohol and measured the optical density. Similar to the data in Figure 2(a), SSE inhibited lipid accumulation in a dose-dependent manner compared with the control group (Figure 2(b)). In parallel assay, intracellular triglyceride content was measured in SSE-stimulated 3T3-L1 adipocytes. Consistent with the results of Oil Red O staining, triglyceride contents were increased significantly in the adipocytes, and SSE treatment decreased the accumulation of triglyceride compared with the differentiated cells (Figure 2(c)). At a maximum concentration of 400 *μ*g/mL, SSE significantly decreased lipid accumulation by up to 56% relative to the MDI-treated positive control group (Figure 2(c)). Similarly, GW9662 treatment used as a positive control markedly reduced triglyceride accumulation in adipocytes.

**Figure 3:**
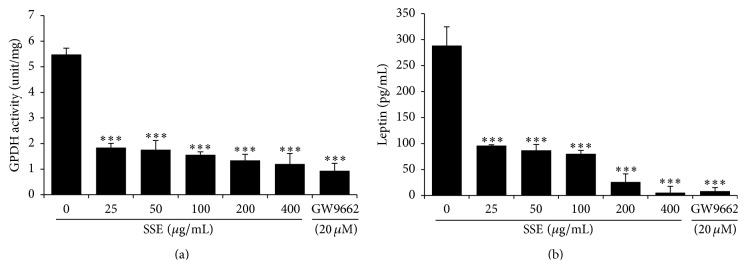
Inhibitory effects of SSE on GPDH activity and leptin production in 3T3-L1 adipocytes. 3T3-L1 preadipocytes were differentiated into adipocytes by incubation with isobutylmethylxanthine, dexamethasone, and insulin (MDI) for 8 days. The cells were exposed to various concentrations of SSE (0, 25, 50, 100, 200, or 400 *μ*g/mL) during the differentiation period. (a) GPDH activity of the cells was assessed by measuring the decrease in NADH at 340 nm using a TAKARA glycerol-3-phosphate dehydrogenase activity assay kit. (b) Culture supernatant was collected from the SSE-treated cells. Leptin production was determined by ELISA by subtracting the value measured at 450 nm using a mouse leptin immunoassay kit (R&D Systems). Data are presented as the mean ± SEM. ^*∗∗∗*^ *p* < 0.01 compared with the differentiated control.

GPDH is an enzyme that generates glycerol-3-phosphate from dihydroxyacetone phosphate in adipocytes for lipid biosynthesis [10]. At a concentration of 25~400 *μ*g/mL, SSE treatment markedly reduced the GPDH activity compared with the cells differentiated into adipocytes (Figure 3(a)). The influence of SSE on the production of leptin, a major adipose hormone [11], was investigated in 3T3-L1 adipocytes. As shown in Figure 3(b), leptin production was inhibited by >50% by SSE treatment at a concentration of 50 to 400 *μ*g/mL.

### 3.3. Effects of SSE on mRNA Expression of Adipogenesis-Related Genes in 3T3-L1 Adipocytes

**Figure 4:**
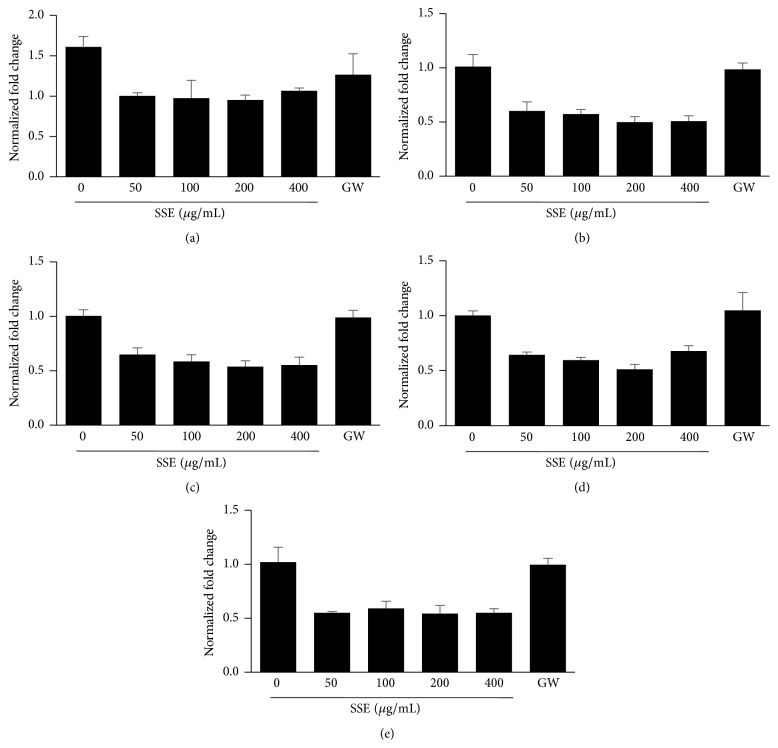
Effects of SSE on mRNA expression of lipid metabolism-related genes in 3T3-L1 adipocytes. (a) 3T3-L1 preadipocytes were differentiated into adipocytes by incubation with isobutylmethylxanthine, dexamethasone, and insulin (MDI) for 6 days. The cells were exposed to various concentrations of SSE (0, 25, 50, 100, 200, or 400 *μ*g/mL) during the differentiation period. Total RNA was isolated and subjected to real-time RT-PCR for* PPAR-γ* (a) and* C/EBP-α* (b),* FAS* (c),* LPL* (d), and* FABP4* (e)*. β-actin* was used as a housekeeping gene.

Adipogenesis is accompanied by changes in the expression of adipogenesis-related transcriptional factors and specific molecular markers [12]. PPAR-*γ* and C/EBP-*α* are major transcription molecules in the adipogenesis pathway [13, 14]. As shown in Figures 4(a) and 4(b), SSE treatment reduced the mRNA expression of* PPAR-γ* and* C/EBP-α* in the differentiated adipocytes. In particular, the SSE caused greater suppression of* PPAR-γ* expression compared with the PPAR-*γ* inhibitor GW9662 [15]. SSE also decreased the expression of PPAR-*γ* target genes* FAS, LPL,* and* FABP4 *in 3T3-L1 adipocytes (Figures 4(c), 4(d), and 4(e)).

### 3.4. Effects of SSE on the Phosphorylation of MAPKs in 3T3-L1 Adipocytes

**Figure 5:**
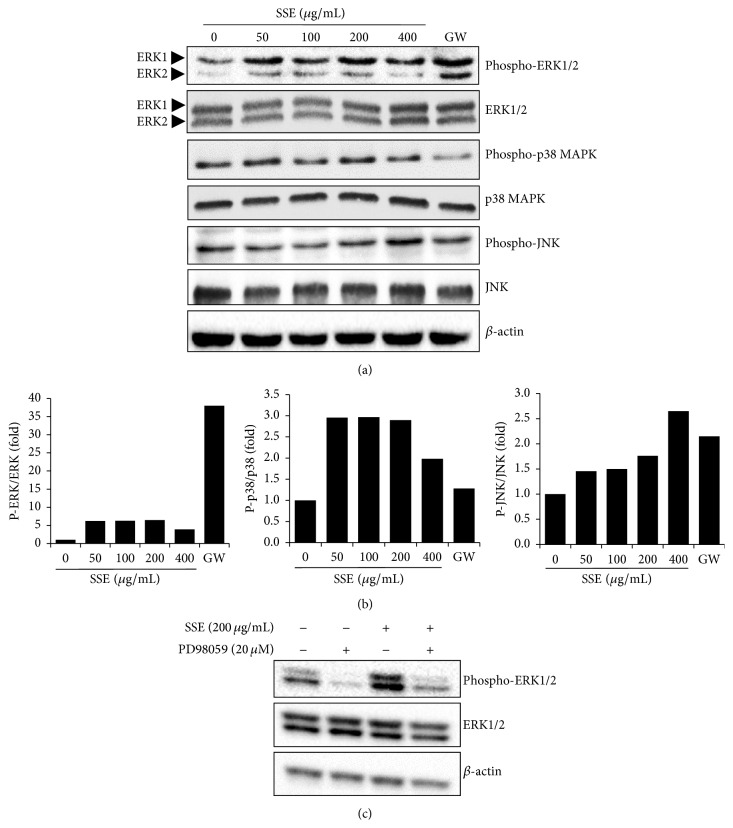
Effects of SSE on phosphorylation of the MAPK family proteins in 3T3-L1 adipocytes. (a) 3T3-L1 preadipocytes were differentiated into adipocytes by incubation with isobutylmethylxanthine, dexamethasone, and insulin (MDI) for 4 days. The cells were exposed to various concentrations of SSE (0, 25, 50, 100, 200, or 400 *μ*g/mL) during the differentiation period. Cell lysates were prepared and subjected to immunoblotting for phospho-ERK1/2, phospho-p38 MAPK, and phospho-JNK. (b) The graph represents the relative levels of phosphorylation of MAPKs. (c) 3T3-L1 cells were treated with SSE and/or ERK inhibitor PD98059. Cell lysates were prepared and subjected to immunoblotting for phospho-ERK1/2.

The MAPK pathways play a role in the regulation of each step in the process of adipogenesis [16]. To examine whether SSE treatment could influence the MAPK pathway, western blotting was conducted using anti-phospho-MAPKs including ERK1/2, p38 MAPK, and JNK. As shown in Figures 5(a) and 5(b), the level of phospho-ERK1/2 was increased by SSE in 3T3-L1 adipocytes. By contrast, SSE treatment had no significant effect on the phosphorylation of p38 MAPK or JNK in the cells. To confirm importance of the ERK pathway in SSE inhibition of adipogenesis, we utilized an ERK inhibitor PD98059 with SSE treatment. As shown in Figure 5(c), cotreatment of SSE and PD98059 blocked SSE-induced phosphorylation of ERK1 in 3T3-L1 adipocytes.

### 3.5. HPLC Analysis of SSE

**Figure 6:**
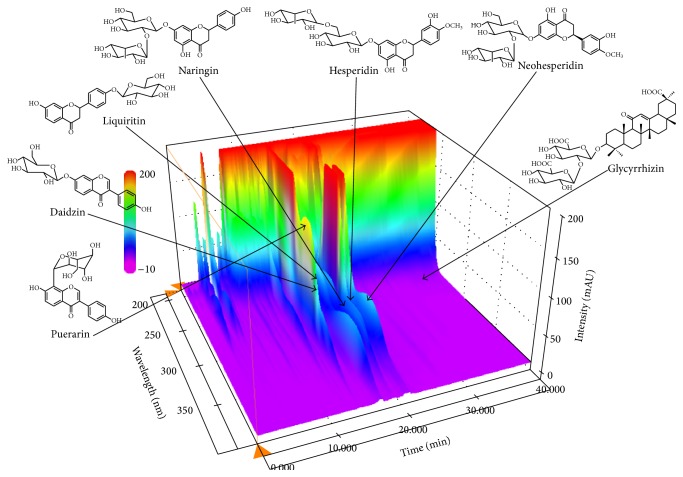
Three-dimensional chromatogram of SSE by HPLC-PDA. HPLC conditions, column: Gemini C_18_ column (250 × 4.6 mm, 5 *μ*m; mobile phase: 1.0% (v/v) acetic acid in water and 1.0% (v/v) acetic acid in acetonitrile; gradient elution: 5–70% B for 0–40 min, 70–100% B for 40–45 min, 100% B for 45–50 min, and 100–5% B for 55 min; flow rate: 1.0 mL/min; column oven temperature: 40°C; injection volume: 10 *μ*L).

We performed the simultaneous determination of seven components for quality control of SSE using HPLC coupled with photodiode array (PDA) detector. The regression equation of each compound was calculated by plotting the peak area (*y*) against the concentrations (*x*, *μ*g/mL) using mixed standard solutions. All calibration curves for quantitative analysis were obtained by assessment of the peak areas from standard solutions in the concentration ranges: puerarin, hesperidin, and neohesperidin, 1.56–200.00 *μ*g/mL; daidzin and liquiritin, 0.78–100.00 *μ*g/mL; naringin, 1.95–250.00 *μ*g/mL; glycyrrhizin, 3.13–100.00 *μ*g/mL. The calibration curves for the 7 compounds showed good linearity (*r* ^2^ ≥ 0.9997). The limit of detection (LOD) and limit of quantitation (LOQ) of the seven investigated compounds were ≤0.52 and 1.74 *μ*g/mL, respectively (Table 3), which indicated that the analytical method was acceptable with satisfactory sensitivity. Using optimized chromatography conditions, three-dimensional chromatogram was obtained using an HPLC-PDA detector (Figure 6). The concentrations of 7 marker compounds were detected from 26 to 10.38 mg/g, and these are summarized in Table 4.

## 4. Discussion

The field of herbal medicine includes the use and study of herbal plants for the purpose of preventing and treating various diseases. Herbs are attractive candidates for new drug development compared with synthetic chemical agents because they elicit fewer adverse effects of herbs. Recent studies have shown that many herbal plants have antiobesity activity by regulating adipogenesis. Lee et al. reported antiobesity effects of Aster glehni extract in both* in vitro* and* in vivo* models [17]. Kwak et al. reported that Aristolochia manshuriensis Kom inhibited adipogenesis by regulating the ERK1/2 and Akt pathways [18]. Kubota et al. reported that Zizyphus jujuba extract blocked adipogenesis by targeting PPAR-*γ* and C/EBP-*α* expression in 3T3-L1 cells [19]. Interestingly, the antiobesity effects of herbal formulas have also been reported in several recent papers [20–23]. An herbal formula is a mixture of several different herbs, which provides a greater efficiency, compared with each herb alone.

In the current study, we found that the traditional Korean herbal formula SSE inhibited adipogenesis in 3T3-L1 cells. Adipogenesis was induced by adding the differentiation stimulators MDI to 3T3-L1 preadipocytes. The differentiated adipose cells exhibited increased triglyceride accumulation, GPDH activation, and leptin production. By contrast, SSE treatment exerted strong inhibitory effects on triglyceride accumulation, GPDH activity, and leptin production in adipocytes (Figures 2 and 3).

Adipogenesis is a multistep process that is regulated by a cascade of various transcription factors. PPAR-*γ* and C/EBP-*α* are key factors in the regulation of the expression of target genes leading to adipocyte development [13, 14, 24]. In our study, SSE markedly decreased the mRNA expression levels of* PPAR-γ* and* C/EBP-α* in 3T3-L1 adipocytes. Consistently, SSE reduced the mRNA expression of the PPAR-*γ* target genes* FAS, LPL,* and* FABP4 *(Figure 4). These data suggest that the principal mechanism responsible for the antiadipogenesis effects of SSE occurs through the inhibition of* PPAR-γ* and* C/EBP-α* expression.

MAPK represents a family of proteins involved in various cellular processes such as cell survival, differentiation, and proliferation [25]. It is known that MAPKs regulate both normal and pathological adipogenesis [16]. ERK1/2 has both negative and positive effects on adipogenesis regulation, whereas p38 MAPK and JNK display limited effects. PPAR-*γ*, C/EBP-*α*, and the ERK pathway have been found to be involved in the regulation of each of adipogenesis [26]. The ERK pathway is necessary for the early proliferative steps of adipogenesis [27]. In addition, Hu et al. reported that the ERK pathway is known to have inhibitory effect on adipocyte maturation by targeting PPAR-*γ* in the later steps of adipogenesis [28]. Consistently, we observed that the herbal formula SSE increased the phosphorylation of ERK in differentiation-induced 3T3-L1 cells. By contrast, other MAPK family members p38 MAPK and JNK were not significantly affected by SSE treatment (Figure 5). Furthermore, cotreatment of SSE and ERK inhibitor PD98059 confirmed importance of the ERK pathway antiadipogenesis of SSE. Together, our data suggest that inhibitory effect of SSE is dependent on the ERK pathway by targeting PPAR-*γ* like Hu et al.'s data [28]. Similar to our study, other studies of natural products including phytochemicals and herbal medicines have reported that their antiadipogenic potential involves targeting of the ERK1/2 pathway [18, 29, 30].

Antiobesity effects have been reported for 9 of the 12 SSE components except for* Angelica decursiva, Poria cocos, and Glycyrrhiza uralensis* [19, 31–37]. For example,* Pueraria lobata* improved impaired glucose and lipid metabolism in obese mice [32].* Pinellia ternata* exhibited antiobesity effects through changes in thermogenesis and fatty acid oxidation [33].* Platycodon grandiflorum* modified adipokines and the glucose uptake in high-fat diet- (HFD-) fed mice and in L6 muscle cells [35]. These findings strongly support the antiobesity activity of SSE and suggest its potential as an antiobesity drug candidate.

HPLC-PDA method is a convenient, widely used, and powerful approach for the rapid identification of constituents in botanical extracts and plants important in traditional Chinese medicine [38]. Hence, in this study, we performed quantitative determination of seven main components in SSE using HPLC-PDA. The investigated components were as follows: puerarin and daidzin form Puerariae Radix, liquiritin and glycyrrhizin from Glycyrrhizae Radix et Rhizoma, and naringin, hesperidin, and neohesperidin from Aurantii Fructus Immaturus and Citri Unshius Pericarpium. The optimized HPLC-PDA method was applied for simultaneous determination of the seven marker compounds in SSE. Among these components, naringin, which is a marker component of Aurantii Fructus Immaturus and Citri Unshius Pericarpium, was detected 10.38 mg/g as the main compounds compared with the others in SSE (Figure 6 and Table 3). The established HPLC-PDA method will be helpful to improve quality control of SSE.

In conclusion, our data demonstrate that SSE has the inhibitory effects on adipogenesis in 3T3-L1 adipocytes as indicated by a significant reduction in triglyceride accumulation without cytotoxicity. The inhibitory effects of SSE may be mediated through the suppression of PPAR-*γ* and C/EBP-*α* as well as activation of the ERK1/2 pathway. Further studies are needed to confirm the antiadipogenesis effects of SSE in obesity using HFD-fed obese animals. Herbal formulas have an advantage for multicompound/multitarget (MCMT) therapy because they are a cocktail drug comprising several different phytochemicals. The antiadipogenic effects of SSE could be expanded to drug development studies for possible treatment of obesity or other metabolic diseases.

## Figures and Tables

**Table 1:** Composition of Samsoeum (SSE).

Herbal medicine	Scientific name	Supplier	Source	Amount (g)
Perillae Folium	*Perilla frutescens*	Omniherb	Geochang, Korea	3.75
Puerariae Radix	*Pueraria lobata*	Omniherb	Jecheon, Korea	3.75
Pinelliae Tuber	*Pinellia ternata*	HMAX	China	3.75
Angelicae Decursivae Radix	*Angelica decursiva*	HMAX	China	3.75
Ginseng Radix Alba	*Panax ginseng*	Omniherb	Geumsan, Korea	3.75
Poria Sclerotium	*Poria cocos*	Omniherb	Yeongcheon, Korea	3.75
Aurantii Fructus Immaturus	*Citrus aurantium*	HMAX	China	2.8125
Platycodonis Radix	*Platycodon grandiflorum*	Omniherb	Yeongcheon, Korea	2.8125
Glycyrrhizae Radix et Rhizoma	*Glycyrrhiza uralensis*	HMAX	China	2.8125
Citri Unshius Pericarpium	*Citrus unshiu*	Omniherb	Jeju, Korea	2.8125
Zingiberis Rhizoma Crudus	*Zingiber officinale*	Omniherb	Yeongcheon, Korea	3.75
Zizyphi Fructus	*Zizyphus jujuba*	Omniherb	Yeongcheon, Korea	3.75

Total amount				41.25

**Table 2:** List of primer sequences for real-time RT-PCR.

Gene	Primer sequences	
*GAPDH*	Forward	5′-ACAATGAATACGGCTACAGCAACAG-3′
	Reverse	5′-GGTGGTCCAGGGTTTCTTACTCC-3′

*PPAR-γ*	Forward	5′-TATGGAGTGACATAGAGTGTGCT-3′
	Reverse	5′-CCACTTCAATCCACCCAGAAAG-3′

*C/EBP-α*	Forward	5′-CAAGAACAGCAACGAGTACCG-3′
	Reverse	5′-GTCACTGGTCAACTCCAGCAC-3′

*FABP4*	Forward	5′-CAAGAACAGCAACGAGTACCG-3′
	Reverse	5′-GTCACTGGTCAACTCCAGCAC-3′

*LPL*	Forward	5′-CTGCTGGCGTAGCAGGAAGT-3′
	Reverse	5′-CTGGAAAGTGCCTCCATTG-3′

*FAS*	Forward	5′-CAAGAACAGCAACGAGTACCG-3′
	Reverse	5′-GTCACTGGTCAACTCCAGCAC-3′

**Table 3:** Regression data, linear range, correlation coefficient, LOD, and LOQ for marker compounds (*n* = 3).

Compound	Linear range (*μ*g/mL)	Regression equation^a^	Correlation coefficient (*R* ^2^)	LOD^b^ (*μ*g/mL)	LOQ^c^ (*μ*g/mL)
Puerarin	1.56–200.00	*y* = 40369.09*x* − 4248.84	0.9997	0.05	0.16
Daidzin	0.78–100.00	*y* = 37991.95*x* + 2862.21	0.9998	0.05	0.17
Liquiritin	0.78–100.00	*y* = 18338.85*x* − 978.63	1.0000	0.06	0.19
Naringin	1.95–250.00	*y* = 17333.29*x* − 2201.40	0.9999	0.06	0.18
Hesperidin	1.56–200.00	*y* = 18669.49*x* − 2370.68	1.0000	0.06	0.18
Neohesperidin	1.56–200.00	*y* = 23891.33*x* − 3109.41	0.9999	0.04	0.14
Glycyrrhizin	3.13–100.00	*y* = 8064.35*x* + 1345.30	0.9999	0.52	1.74

^a^ *y*: peak area (mAU) of compounds; *x*: concentration (*μ*g/mL) of compounds.

^b^LOD = 3 × signal-to-noise ratio.

^c^LOQ = 10 × signal-to-noise ratio.

**Table 4:** Contents of seven compounds in the SSE by HPLC (*n* = 3).

Compound	Mean (mg/g)	SD	RSD (%)	Source^*∗*^
Puerarin	5.29	0.01	0.22	PR
Daidzin	1.26	0.01	1.15	PR
Liquiritin	2.27	0.03	1.20	GRR
Naringin	10.38	0.05	0.49	AFI, CUP
Hesperidin	5.64	0.05	0.87	AFI, CUP
Neohesperidin	6.01	0.11	1.80	AFI, CUP
Glycyrrhizin	3.96	0.04	0.97	GRR

^*∗*^PR: Puerariae Radix, GRR: Glycyrrhizae Radix et Rhizoma, AFI: Aurantii Fructus Immaturus, and CUP: Citri Unshius Pericarpium.
